# Changes in Serum Urate Levels after Bariatric Surgery in Patients with Obesity: An Observational Study

**DOI:** 10.1007/s11695-024-07191-8

**Published:** 2024-03-25

**Authors:** Daniel W. Mills, Dylan M. Woolley, Basil J. Ammori, Hector Chinoy, Akheel A. Syed

**Affiliations:** 1 General (Internal) Medicine, Salford Royal Hospital, Northern Care Alliance NHS Foundation Trust, Salford, UK; 2 Metabolic and Bariatric Surgery, Salford Royal Hospital, Northern Care Alliance NHS Foundation Trust, Salford, UK; 3 Metabolic and Bariatric Surgery, Burjeel Hospital, Burjeel Holdings, Abu Dhabi, UAE; 4 Faculty of Biology, Medicine and Health, The University of Manchester, Manchester, UK; 5 Rheumatology, Salford Royal Hospital, Northern Care Alliance NHS Foundation Trust, Salford, UK; 6 Diabetes, Endocrinology and Obesity Medicine, Salford Royal Hospital, Northern Care Alliance NHS Foundation Trust, Salford, UK

**Keywords:** Hyperuricaemia, Uric acid, Weight loss, Gastric bypass, Gout

## Abstract

**Background:**

Obesity is a risk factor for hyperuricemia and gout, while weight reduction can reduce urate levels. The aim of this study was to examine the effect of bariatric surgery on longitudinal serum urate levels.

**Methods:**

We performed a retrospective observational study of 283 patients who had undergone bariatric surgery [237 (83.7%) gastric bypass, 34 (12.0%) sleeve gastrectomy and 12 (4.2%) gastric banding] and were followed up for 2 years. The results shown represent mean (standard deviation).

**Results:**

Bariatric surgery was associated with significant reduction in serum urate from baseline level of 0.343 (0.086) mmol/L to 0.296 (0.076) mmol/L (*p* < 0.001) at 12 months and 0.286 (0.073) mmol/L (*p* < 0.001) at 24 months, including in men and women, and in patients with or without diabetes. Patients with elevated urate levels at baseline, who comprised 27.2% of the total cohort, achieved reduction in levels by 4 months.

**Conclusion:**

Bariatric surgery leads to significant reduction in serum urate levels at 12 and 24 months. This could reduce incidence of gout and need for prophylactic medication(s).

**Graphical Abstract:**

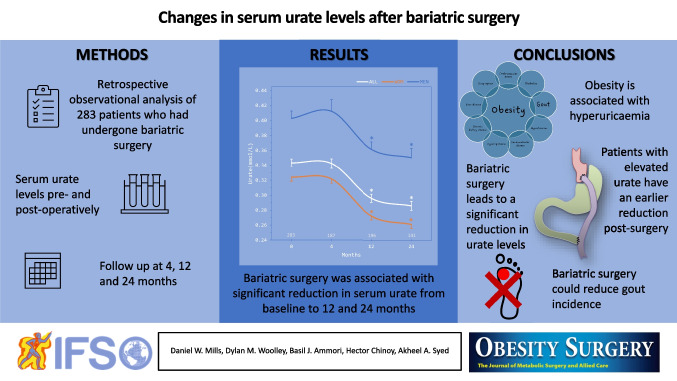

## Introduction

Obesity is a risk factor for hyperuricemia [1, 2], which can cause gout [3, 4], uric acid renal disease, chronic kidney disease, hypertension, and cardiovascular disease [5, 6]. Weight loss is known to be effective in reducing urate levels [7, 8], thereby reducing incidence of gout and associated diseases. A large systematic review found that greater weight loss results in a greater reduction in serum uric acid levels at medium-to-long term follow-up [7].

Bariatric surgery, the most effective and durable weight loss intervention, reduces rates of metabolic and obesity-associated diseases [9, 10]. However, bariatric surgery has been associated with temporarily increased serum uric acid levels and gout attacks in the immediate postoperative period in a study of 60 patients with type 2 diabetes undergoing sleeve gastrectomy [11], and another study of 99 patients with pre-existing gout who underwent bariatric surgery compared to patients undergoing non-surgical weight management [12].

We therefore studied the effect of bariatric surgery on longitudinal serum urate levels in a larger sample of patients with obesity. We sought to examine the rate of postoperative reduction in serum urate levels categorized by presence or absence of preoperative hyperuricemia defined by sex-specific cut-off levels, and by presence or absence of diabetes.

## Methods

We performed a retrospective observational analysis of patients who had undergone bariatric surgery in a National Health Service (NHS) university teaching hospital in Northwest England. We identified patients for the current study from a pre-existing database of patients who had undergone bariatric surgery over a three-year period [13]. Patients were followed up over a two-year period at 4, 12, and 24 months, and were included if they had a minimum of two serum urate measurements, one pre- and one post-operative level. A total of 283 patients who met the inclusion criteria were included in the study. Data collected included urate levels alongside demographics and obesity-associated disease data including sex, weight, height, blood pressure, HbA1c, and presence of hypertension and diabetes. The reference ranges for serum urate were 0.2–0.43 mmol/L for males and 0.14–0.36 mmol/L for females. We performed descriptive statistical analysis of demographic data, reported as mean ± standard deviation (SD). Comparative analyses between groups were computed with the Student *t* test. Significance level was set at *p* < 0.05. IBM SPSS Statistics 25.0 (IBM Corp, Armonk, NY) was used for analysis.

## Results

Of the 283 patients in the study, 214 (75.6%) were women and 69 (24.4%) were men with mean (SD) age of 45.7 (10.3) years (Table 1). At baseline, 104 (36.7%) had diabetes, 126 (44.5%) hypertension, 62 (21.9%) hyperlipidemia and 92 (32.5%) obstructive sleep apnea treated with CPAP. The bariatric procedures included 237 (83.7%) gastric bypass, 34 (12.0%) sleeve gastrectomy and 12 (4.2%) gastric banding.

**Table 1:** Baseline characteristics of all participants and categorized by sex and presence or absence of diabetes

	All *n* = 283	Women *n* = 214	Men *n* = 69	DM *n* = 104	No DM *n* = 179
Age (yr)	45.7 (10.3)	45.1 (10.2)	47.8 (10.5)	50.2 (9.1)	43.1 (10.2)
Weight (kg)	139.5 (24.8)	134.5 (21.9)	155.2 (26.9)	139.2 (25.0)	139.7 (24.8)
BMI (kg/m^2^)	51.1 (7.1)	51.2 (7.0)	50.8 (7.5)	50.3 (7.6)	51.6 (6.9)
HbA1c (mmol/mol)	51.8 (20.8)	49.8 (20.4)	57.1 (21.1)	65.7 (21.2)	39.2 (9.3)
Systolic BP (mmHg)	147.7 (20.1)	146.6 (20.4)	151.1 (19.1)	146.6 (20.3)	148.3 (20.1)
Diastolic BP (mmHg)	89.9 (13.2)	89.9 (13.0)	89.9 (13.8)	88.7 (14.5)	90.6 (12.4)
Urate (mmol/L)	0.343 (0.086)	0.324 (0.078)	0.403 (0.083)	0.346 (0.094)	0.341 (0.081)

Data shown is mean (standard deviation)

*DM* diabetes mellitus, *BMI* body mass index, *BP* blood pressure

**Fig. 1:**
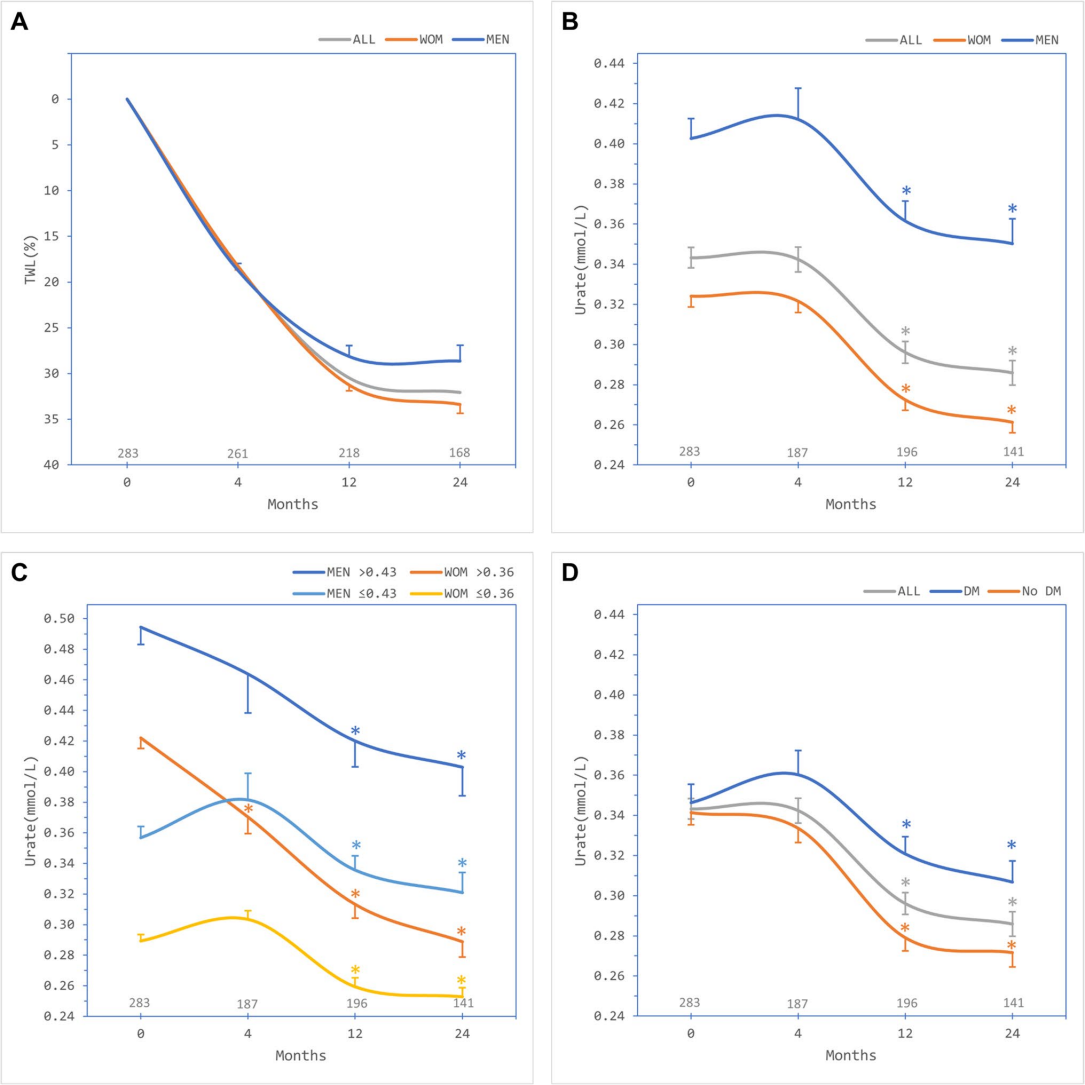
Outcomes after bariatric surgery over 24 months of follow-up. **A** Mean percent total weight loss (TWL) overall (ALL, grey trace) and in males (MEN, blue) and females (WOM, orange). **B** Mean serum urate levels overall (ALL, grey) and in males (MEN, blue) and females (WOM, orange). **C** Mean serum urate levels in males with baseline urate levels > 0.43 mmol/L (MEN > 0.43, blue) or ≤ 0.43 mmol/L (MEN ≤ 0.43, teal), and females with baseline urate levels > 0.36 mmol/L (WOM > 0.36, orange) or ≤ 0.36 mmol/L (WOM ≤ 0.36, yellow). **D** Mean serum urate levels overall (ALL, grey) and in patients with diabetes (DM, blue) or without diabetes (No DM, orange). Numbers of patients contributing data at each timepoint depicted above the x-axis. Error bar, standard error of the mean. *Significant change compared to respective baseline, paired Student *t* test

Mean (SD) baseline weight was 139.5 (24.8) kg and BMI 51.1 (7.1) kg/m^2^. There was significant weight reduction over 24 months (Fig. 1A). Mean (SD) baseline serum urate was 0.343 (0.086) mmol/L. Overall serum urate levels were unchanged at 4 months but there was significant reduction to 0.296 (0.076) mmol/L (*p* < 0.001) at 12 months and 0.286 (0.073) mmol/L (*p* < 0.001) at 24 months compared to baseline, including in men and women (Fig. 1B).

Of the 283 patients in the study, 77 (27.2%) had baseline urate levels greater than the sex-specific cut-offs, including 56 of 214 women (26.2%) and 21 of 69 men (30.4%). Patients with baseline urate levels that were higher than the sex-specific cut-offs had reduction in levels evident by 4 months which achieved statistical significance in women (Fig. 1C), whilst those with lower baseline levels had unchanged levels at 4 months with significant reductions becoming evident from 12 months.

Patients with diabetes had significant but smaller reductions in serum urate levels at 12 and 24 months compared to patients without diabetes (Fig. 1D).

## Discussion

We report significant reductions in serum urate levels universally in both men and women and in patients with or without diabetes within 12 months of bariatric surgery and sustained at 24 months. In patients at the highest risk of gout—those with elevated baseline urate levels above the sex-specific upper limit of normal—a rapid reduction in levels was seen at the four-month follow-up timepoint.

There was a significant reduction in urate levels in patients with or without diabetes. The smaller reduction seen in patients with diabetes could be explained by the effect of insulin resistance on urate levels. Increased insulin resistance is known to decrease urate clearance by the kidneys and so leads to increased urate levels and is a risk factor for hyperuricemia [3, 5]. Increased blood glucose levels can also be associated with increased urate levels [14].

Intriguingly, for patients whose levels were within the normal range preoperatively, serum urate levels did not significantly change at four months despite significant weight loss. Considering reasons for this, we can look at how weight loss is thought to affect urate levels. Initial periods of starvation and catabolism lead to increased rate of hyperuricemia which in turn increases the risk of gout, as shown in a recent meta-analysis [15]. Another consideration is renal function as it influences urate homeostasis [3]. Renal dysfunction associated with major bariatric surgery may contribute to the initial increases in serum urate [16, 17]. Although beyond the scope of this study, it may be interesting in further research to examine how urate levels post-bariatric surgery correlate to renal function or if there is any difference in patients experiencing postoperative acute kidney injury to those who don’t.

Previous studies reported similar reduction in serum urate levels [18, 19], and fewer episodes of gout in association with weight loss after bariatric surgery [15], while some promoted bariatric surgery as a treatment approach for gout in patients with obesity [18, 20]. Other studies looking at the pattern of serum urate levels and time post-bariatric surgery also showed an initial increase in urate levels post-bariatric surgery followed by reduction to below preoperative levels. The period during which urate levels seem to increase varies in different studies. It ranges from 1 to 6 months, with levels decreasing at 12 months after bariatric surgery [7, 15].

Our study offers new insights by highlighting a difference in those patients who have preoperative hyperuricemia vs normal levels and the rate of postoperative reduction in serum urate levels. Given hyperuricemia is the main risk factor for gout [3], a reduction in urate levels can be expected to result in a reduction in gout attacks across a patient population. Many patients with hyperuricemia, however, will never suffer from gout. Nonetheless, bariatric surgery—by effectively lowering urate levels—is likely to reduce the longer-term risk of gout attacks in patients with obesity.

## Limitations

Our study was limited by the retrospective design and small numbers in some subgroups which restricted some analyses. It was also beyond the scope of this study to evaluate frequency of gout attacks and use of urate-lowering medical therapies before and after bariatric surgery. Nonetheless, we offer new insights by looking at patients with and without diabetes and demonstrating a difference in the rate of postoperative reduction in serum urate levels between patients with preoperative hyperuricemia vs normal levels.

## Conclusion

Bariatric surgery significantly reduces urate levels in patients with obesity, either with or without diabetes. Reduction in urate levels to below baseline levels is seen by 12 months at the latest, with further reductions observed at 24 months. In patients with hyperuricemia, who are at higher risk of gout, a quicker reduction in serum urate levels is seen within 4 months with a similar sustained reduction at further follow-up. We conclude that bariatric surgery is effective in lowering serum urate levels and thereby likely to reduce the longer-term risk of gout attacks in patients with obesity.
